# Early and Late Mortality Predictors in Patients with Acute Aortic Dissection Type B

**DOI:** 10.1155/2022/7869356

**Published:** 2022-11-26

**Authors:** Ratko M. Lasica, Jovan P. Perunicic, Dejana R. Popovic, Igor B. Mrdovic, Ross A. Arena, Nebojsa L. Radovanovic, Mina R. Radosavljevic-Radovanovic, Lazar D. Djukanovic, Milika R. Asanin

**Affiliations:** ^1^Emergency Hospital, University Clinical Center Serbia, Pasterova 2, Belgrade 11000, Serbia; ^2^Division of Cardiology, University Clinical Center Serbia, Visegradska 26, Belgrade 11000, Serbia; ^3^Department of Physical Therapy, College of Applied Health Sciences, University of Illinois Chicago, 1200W Harrison St, Chicago, IL 60607, USA

## Abstract

**Background/Aim:**

Despite technological advances in diagnosis and treatment, in-hospital mortality with acute aortic dissection type B is still about 11%. The purpose of this study was to assess the risk factors for early and long-term adverse outcomes in patients with acute aortic dissection type B treated medically or with conventional open surgery.

**Methods:**

The present study included 104 consecutive patients with acute aortic dissection type B treated in our Center from January 1^st^, 1998 to January 1^st^, 2007. Patient demographic and clinical characteristics as well as in-hospital complications were reviewed. Univariate and multivariate testing was performed to identify the predictors of in-hospital (30-day) and late (within 9 years) mortality.

**Results:**

92 (88.5%) patients were treated medically, while 12 (11.5%) patients with complicated acute aortic dissection type B were treated by open surgical repair. In-hospital complications occurred in 35.7% patients, the most often being acute renal failure (28%), hypotension/shock (24%), mesenteric ischemia (12%), and limb ischemia (8%). The in-hospital mortality rate was 15.7% and the 9-year mortality rate was 51.9%. Independent predictors of early mortality in patients with acute aortic dissection type B were uncontrolled hypertension (HR-20.69) and a dissecting aorta diameter >4.75 cm (HR-6.30). Independent predictors of late mortality were relapsing pain (HR-7.93), uncontrolled hypertension (HR-7.25), and a pathologic difference in arterial blood pressure (>20 mmHg) (HR-5.33).

**Conclusion:**

Knowledge of key risk factors may help with a better choice of treatment and mortality reduction in acute aortic dissection type B patients.

## 1. Introduction

The term “acute aortic syndrome” refers to a group of catastrophic diseases that affect the aorta [1, 2]. The incidence of AAD in the Oxford Vascular study is estimated at six per hundred thousand persons per year [3]. According to population-based longitudinal study results, 21% of these patients die before hospitalization [4]. While hourly mortality data for AADB are not available, the overall in-hospital mortality is reported to be 11%, and in the highest risk group mortality can be as high as 71% [5]. The Global Burden Disease 2010 project demonstrated that the overall global death rate from aortic aneurysms and AAD increased from 2.49 per 100 000 to 2.78 per 100 000 between 1990 and 2010, with higher rates for males [6, 7]. AAD is correctly diagnosed in only 15%–43% of patients during their initial presentation [8]. The choice for diagnostic imaging depends on the patient's stability, local expertise, and availability. Computed tomography (CT) of the chest is the preferred first diagnostic imaging method in hemodynamically stable patients [9], transesophageal echocardiography (TEE) is preferred in hemodynamically unstable patients [10], and transthoracic echocardiography is the most frequently used technique for determining diameters of proximal aortic segments in clinical practice [11].

The majority of AADB patients (“uncomplicated dissection”) are managed medically [12–14]. The term “complicated” AADB is used for patients who present with rupture, malperfusion syndromes, or refractory pain [15]. Patients with complicated AADB usually require immediate endovascular or open surgical intervention.

According to the literature, besides direct markers of malperfusion or aortic rupture (renal insufficiency, hypotension, and shock), the most common independent predictors of both in-hospital and late mortality in patients with AADB are female gender, a previously diagnosed aortic aneurysm, a history of atherosclerosis, false lumen patency, a presence of pleural effusion found by heart and lung radiography, and >70 years of age [15].

Since mortality predictors in patients with AADB still required further investigation, the current study analyzes and identifies early and late predictors of mortality in patients with AADB. We hypothesized that it is possible to find independent mortality predictors in patients with AADB. This will help to better guide treatment and reduce mortality in these patients.

## 2. Methods

In this prospective, observational study, we analyzed all patients with AADB (classic dissection) treated at our Institute from January 1^st^, 1998 to January 1^st^, 2007 (*n* = 104). The study was approved by the local hospital ethics committee. Informed verbal and written consent was obtained from all patients before enrollment. The study was performed in accordance with the Helsinki Declaration. All demographic characteristics of patients as well as their signs and symptoms on presentation were recorded (Table 1). Initial pain was identified prospectively on presentation or retrospectively as recoded in our surgical, pathological, and echocardiographic databases. Pain was assessed subjectively by the patient on a numerical scale from 1 to 10 (from 1 to 3 - mild pain; from 4 to 6 - moderate pain; from 7 to 9 severe pain; 10 - worst possible pain). The presence of relapsing pain in the first 24 hours of hospitalization and its significance in the prediction of a poor outcome were particularly observed. Unbearable pain is uncontrolled pain of great intensity that is persistent or recurrent despite aggressive medical treatment. We monitored the exact time and time period of AAD initial pain occurrence as well as monthly and seasonal variability. We paid special attention to dissection risk factors (previous hypertension, smoking, hyperlipidemia, diabetes, atherosclerosis, Marfan syndrome, already existing aortic aneurysm, and positive family history). For hypertension at reception, the systolic blood pressure value is higher than 149 mmHg, and for hypotension, the systolic blood pressure value is less than 100 mmHg. The presence of a pathologic difference in arterial blood pressure measured in both upper extremities; a blood pressure difference over 20 mmHg was considered pathologic. Especially monitored is the presence of uncontrolled hypertension, which is defined as a systolic arterial blood pressure value above 120 mmHg in the first 24 hours of hospitalization despite the use of maximum antihypertensive therapy. The presence of syncope at the initial presentation was observed. Malperfusion, that is, end-organ ischemia caused by branch-vessel involvement and resulting in clinical symptoms of organ damage (heart-myocardial necrosis; brain-various clinical presentations reproducing ischemic patterns; spine-consequent paraplegia; bowel-mesenteric ischemia; kidneys-renal insufficiency and severe refractory hypertension; lower limb-acute ischemia) was also monitored. Malperfusion was followed by an increase in ischemia markers, liver enzymes, a rise in nitrogen matter, and finally metabolic acidosis. The presence of intrahospital complications (large neurological deficit/coma, hypotension/shock, aortic branches being affected by dissection, acute renal insufficiency, mesenteric ischemia, and lower limb ischemia) were also monitored. Their presence at reception and their development in the first 24 hours are especially monitored. Patients with laboratory registered progressive azotemia (at least a double and/or higher increase in blood urea and creatinine) and impaired regulation of water and electrolytes (metabolic acidosis, hyperkalemia) with reduced urine excretion (oliguria or anuria) had acute renal failure. The presence of renal failure on admission was observed (significantly in patients who did not previously have it) and especially during the first 24 h hospitalization. The occurrence of renal failure in this period was considered in order to estimate its prediction of a poor outcome. Risk factors in deceased and living patients were compared.

Acute type B aortic dissection was defined as any nontraumatic dissection that involved the descending aorta and that appeared within 14 days of symptom onset. Diagnosis of AAD was based on anamnestic data, and clinical signs and symptoms, as well as positive results by the diagnostic methods (TTE positive in 81/103 (78.6%), TEE positive in 29/32 (90.6%), CT positive in 41/47 (87.2%), MRI positive in 13/13 (100%), and aortography positive in 67/67 (100%)). Diagnostic methods were used also to define the width of the dissecting aorta in the widest part of the particular segment: diagnostic cut-off values were >4.75 cm for the descending aorta and >3.35 cm for the abdominal aorta, respectively. The absence of false lumen thrombosis (patent false lumen) was especially monitored with CT, and TEE was used in cases where CT was not possible for any reason.

Treatment of patients (medical and open surgical repair) and their survival in function of the type of treatment were also monitored. As part of the initial drug therapy in our patients, opioid analgesics were used to relieve pain (morphine sulfate in a dose of 4–8 mg i.v. at intervals of 5–15 min. until the pain subsides). Beta blockers were used as first-line drugs to reduce stress on the aortic wall (metoprolol in an initial dose of 5 mg i.v. for 5 min. and then in a maintenance dose of 5–10 mg i.v. every 4–6 hours; or esmolol in a dose of 0.5 mg/kg for 2–5 min. followed by an infusion of 0.1–0.2 mg/kg/min.). Patients who did not tolerate beta blockers were treated with non-dihydropyridine calcium channel antagonists (verapamil, 5 mg i.v. every 5–10 min.). In patients with extreme hypertension who were previously given beta blockers, vasodilators were used (Na-nitroprusside in an initial dose of 0.25 *μ*gr/kg/min., with titration to maintain a mean arterial blood pressure of 60–70 mmHg). Intravenous administration of nitroglycerin was also used in some patients in order to reduce the value of arterial blood pressure (initial dose 5 *μ*gr/min. i.v. infusion with an increase of 5 *μ*gr every 3–5 minutes up to a maximum of 200 *μ*gr/min). For further control of arterial blood pressure and heart rate, beta blockers, ACE inhibitors, and angiotensin receptor blockers were administered orally in adjusted doses for each patient. Adherence was maintained. Surgery was indicated in patients with: (1) unbearable pain (durable and repeated); (2) with uncontrolled hypertension; (3) sudden increase of aorta diameter; (4) occurrence of periaortic or mediastinal hematoma (signs of threatening aortic rupture); and (5) presence of malperfusion signs (ischemia of limbs, intestines, kidneys, CNS malperfusion signs). In-hospital (30-day) and late (within 9 years) survival of patients as a function of treatment applied were also analyzed.

Patients were invited by telephone for follow-up clinical examinations after discharge from the hospital. Examinations were performed after 1 month, 3 months, 6 months, and after one year of discharge, with regular annual controls.

### 2.1. Statistical Analysis

Variables were reported either as a percentage or the mean ± standard deviation. Statistical analyzes were performed using the SPSS 17.0 software. The unpaired Student's *t*-test, *χ*^2^ test and Mann–Whitney *U* test were used for continuous and categorical variables. A *p* value <0.05 was considered statistically significant. The risk of type I error was therefore 0.05 for each individual comparison. For assessing the predictive risk factors for mortality, a Cox proportional hazards analysis was performed using the forward stepwise procedure. Each variable that was considered significant at univariate analysis was selected for multivariate analysis (method: likelihood ratio). The variables were added one at the time to the model if they met the selection criterion based on the *p* value for the score statistics. The default value for inclusion was 0.1. Kaplan–Meier cumulative survival plots and Log Rank test were used for the survival among groups comparison. The ROC curve (receiver operating characteristic Curve) was used to determine cut-off values of examined parameters.

## 3. Results

### 3.1. Circadian Analysis

Pain, as the main AAD symptom, occurred suddenly in 94 (91.3%) patients. Pain occurred in a.m. hours (midnight to noon) in 73.3% patients, while it occurred in the p.m. hours (noon to midnight) in 26.6% patients. The occurrence of initial pain from 6 a.m. to noon was statistically more frequent than in the remaining three 6-hour periods (*χ*^2^ = 23.656; *p* < 0.001). The AADB peak was from 8 to 9 a.m. Initial pain occurred in patients with AADB on Wednesdays and Fridays (by 20%), more frequently in the winter months (especially February-18.8%) than within the other three seasonal periods (*χ*^2^ = 18.195, *p* < 0.001).

### 3.2. Treatment and In-Hospital Complications in AADB Patients

More patients in our study were treated medically than surgically (88.5% vs. 11.5%). Initial medical treatment most frequently included beta blockers (85.7%). Most frequent indications for proceeding a patient proceeding to urgent surgery were malperfusion (5/12 or 41.7% (kidney malperfusion with signs of acute renal insufficiency was diagnosed in three patients; one patient had mesenteric ischemia; one patient had monoplegia)), threatening aortic rupture (3/12 or 25%), limb ischemia (1/12 or 8.3%) and persistent pain (1/12 or 8.3%). The other two patients were operated on because of an urgent indication in AADB as well as two or more indications for surgery: (1) one (8.3%) was operated because of durable and repeated pain and kidney malperfusion signs (with the occurrence of acute renal insufficiency); and (2) one (8.3%) was operated because of durable and repeated pain, kidney malperfusion (acute renal insufficiency), and signs of threatening aortic rupture.

In-hospital complications occurred in 35.7% patients: acute renal insufficiency (28%), hypotension/shock (24%), mesenteric ischemia (12%), and aortic branches being affected (8%).

### 3.3. Early Survival

**Figure 1:**
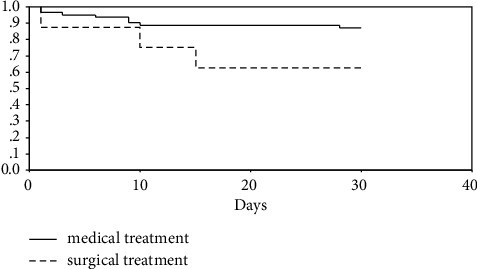
Kaplan–Meier's survival curve in function of treatment in aortic dissection type B.

In-hospital mortality in the cohort studied was 15.7% (16 of 104), 82% of which died within the first ten hospitalization days. All patients deceased within these first thirty days were males (*p*=0.014); 90.9% of them were not treated for prior hypertension (*p* < 0.001). Comparing deceased and surviving patients, pathological difference in blood pressure was present in 54.5% vs. 25.4%; *p*=0.041; syncope in 54.5% vs. 16.9%; *p*=0.006; relapsing pain in 90% vs 25.4%; *p* < 0.001; monoplegia/monoparesis was present in 27.3% vs. 6.8%, *p*=0.034 and patent false lumen in 90.9% vs. 49.2%, *p*=0.040, respectively. In-hospital mortality was higher in patients treated surgically compared to those treated medically (37.5% vs 12.9%) (Figure 1).

Univariant Cox regression analysis showed that the most important predictors of early mortality in patients with AADB are the presence of intrahospital complications (HR-21.2, 95% CI 2.71–16.9; *p*=0.003), relapsing pain (HR-20.4, 95% CI 2.5–16.16, *p*=0.004) and uncontrolled hypertension (HR-13.9; 95% CI 2.9–64.5; *p*=0.001). The multivariate Cox analysis showed that independent predictors of early mortality are uncontrolled hypertension and the maximum diameter (>4.75 cm) of the dissecting aorta (Table 2).

### 3.4. Overall Survival

The overall mortality in this study was 54 of 104 (51.9%), 90% of which were males; *p*=0.013. Syncope on admission (45% vs 14%; *p*=0.016), relapsing pain (94.7% vs 12%; *p* < 0.001), monoplegia/monoparesis (20% vs 6%; *p*=0.023), patent false lumen (85% vs 44%; *p*=0.172), aortic branches being affected (40% vs 7.4%; *p*=0.022), intrahospital complications (80% vs 18%; *p* < 0.001), and uncontrolled hypertension (70% vs 13%; *p* < 0.001) were most frequent in the deceased.

Long-term follow-up showed higher mortality in surgically treated patients, but this rate did not increase compared to the previous follow-up period (37.5%); on the other hand, mortality in medically treated patients increased to 27.4%. Mortality rates were higher in surgically treated patients both in early and overall survival. Long-term follow-up also showed that most patients died from cardiovascular conditions: aortic dissection 63.2%, cerebrovascular insult 10.5%, and acute myocardial infarction 10.5%.

Univariant Cox regression analysis showed that the most important predictors of overall mortality in patients with AADB are relapsing pain (HR-49.04; 95%CI 6.50–37.00; *p* < 0.001) and the presence of intrahospital complications (HR-8.88; 95% CI 2.95–26.65; *p* < 0.001). The multivariant Cox analysis showed that independent predictors of overall mortality in patients with AADB are relapsing pain (HR- 7.93; 95% CI 1.00–62.87; *p*=0.050), uncontrolled hypertension (HR-7.25; 95% CI 1.89–27.65; *p*=0.003), and arterial blood pressure difference over 20 mmHg (HR 5.33; 95% CI 1.45–19.67; *p*=0.012) (Table 3).

## 4. Discussion

This report indicates a strong influence of rhythmicity on the occurrence of AADB, the importance of early diagnosis of in-hospital complications, one of the leading causes of mortality in patients with AADB, and demonstrates the predictors of both early and late mortality.

Peak incidence of AADB in our patients occurred during the early morning, when individuals awaken and begin their activities (peak between 6 a.m. and noon, 53.3%), most frequently between 8 and 9 in the morning, which corresponds to the findings by Lasica et al. [16]. The frequent occurrence of AAD in the early morning hours is related with a surge in sympathetic activity, leading to an increase in shear forces secondary to elevation in blood pressure, heart rate, and the rate of pressure change (dP/dt) [17, 18]. With regards to seasonal variation, we recorded most AAD onset cases in the winter months, especially February; this was confirmed also by large studies assessing acute myocardial infarction, sudden cardiac death, and cardiac arrests [16, 18, 19].

Both our and IRAD studies showed a similar number of AADB patients with previously poorly treated hypertension (94.3% vs. 76.7%), diabetes mellitus (8.4% vs. 6.6%), diagnosed aortic aneurysm (8.6% vs. 2.2%), and Marfan syndrome (1.4% vs. 1.8%) [5]. The most common risk factor associated with AADB is hypertension, observed in 65–75% of individuals and mostly poorly controlled [20–22]. Our patients were most commonly hypertensive on admission, while the number of those who were hypotensive or in shock was much lower (85.6% vs 1.4% vs 1.4%, respectively).

In our study, 99% of patients had pain (occurred suddenly in 91.3%), mostly localized in the chest (42.7%). Initial pain in AADB had a migratory character in 81% of cases. Early diagnosis and adequate treatment were most important factors for a favorable outcome with aortic dissection [23]. It is sometimes very difficult to diagnose aortic dissection because AAD is not followed by pain in 5–17% of cases [5, 24, 25].

Both our results and the IRAD study results show that patients with AADB are usually treated medically (88.5% vs 78% respectively; 11.5% vs 20% respectively). Indeed, patients with uncomplicated AADB should be treated medically (with antihypertensive drugs) in the acute stage. The overall goal in the acute management of dissection is blood pressure reduction and minimizing ΔP/ΔTmax on the aortic wall [9]. *β*-blockers are the first-line drug of choice (applied in 85.7% our patients) because they control the maximal force of left ventricular contraction (dP/dt_max_) in addition to controlling heart rate and arterial blood pressure.

Specific initial findings that are indications for emergency surgery (rupture, shock, organ ischemia) negatively affect the course of AADB. A number of studies showed that surgical mortality is much higher in the acute stage (even around 35–75%) than in the chronic stage of the disease, and also that late mortality is higher in patients treated medically than surgically [21, 26]. Mortality in patients treated surgically cannot be attributed to the form of treatment but rather a complicated disease course in patients undergoing surgery. In fact, surgical patients are, in general, those who have the factors that negatively affect survival rate. In view of the foregoing selection of optimal therapy, it should individually depend on the condition of the patient.

When comparing the results of our study with those of Genoni et al. [21], malperfusion was a more frequent indication for emergency surgery in our patients (34% vs. 41.7%) and aortic rupture as an indication was present in approximately the same percentage of patients (27% vs. 25%). While rupture diagnosis is clinically easily recognizable, malperfusion is very difficult to be diagnosed despite the fact that it occurs in over 30% of AAD patients [21, 27]. Undiagnosed visceral malperfusion in the acute stage of disease is usually the reason for an increased mortality rate in medically treated patients.

In our study and in the study of Shih-Hung Chan et al. [28], intrahospital mortality was higher in patients treated surgically (37.5% vs. 12.9% and 43.5% vs. 9.8%). Mortality was higher in the group of surgically treated patients and in IRAD (32% vs. 10%). Therefore, compared with medically treated patients, surgical patients are at high risk, and it would be an error to compare the survival of these two patient groups.

The long-term prognosis for patients with AADB is variable and exceeds the cumulative incidence of mortality in other diseases such as coronary artery disease and moderate chronic obstructive pulmonary disease [15]. Our nine-year survival rate was of 48.1% which is comparable with the results of the other studies [2, 15, 29]. Late mortality was higher in our study among surgically treated patients (37% vs. 27.4%). However, it was noted that mortality among these patients did not rise after 30 days of treatment while it rose among medically treated ones, which may be explained by possible unrecognizing already existing malperfusion or progression of aortic aneurysmatic dilatation [30].

Our results showed the presence of relapsing pain, false lumen patency, and the existence of syncope on admission to be important for a poor intrahospital outcome in patients with AADB [15, 29, 31]. The presence of intrahospital complications (acute renal insufficiency, hypotension/shock, mesenteric ischemia, and aortic branches affected by dissection) in acute stage AADB is the most important predictor of intrahospital mortality in patients with AADB. The results of our study coincide with the results of previous research that demonstrates uncontrolled hypertension and an increased diameter of the dissecting descendent aorta (>4.75 cm in our study) are among the most important predictors of early mortality in patients with AADB [15, 27, 32]. Age (over 70) and female gender were not considered independent mortality predictors in our study, while some authors considered them as an important mortality predictors in the AADB [15]. We posit that relapsing pain during hospitalization is an important independent predictor of late mortality in patients with AADB patients while prior uncontrolled hypertension is confirmed as a known independent predictor of late mortality [15, 31].

### 4.1. Study Limitations

Endovascular procedures (fenestration or stent grafting) were not considered in the current patient cohort because of technical impossibilities and the team's inadequate level of training. Since the study was carried out in a single medical, the number of individuals examined was not large enough to obtain more precise data, which would surely be possible with a larger cohort.

## 5. Conclusion

Initial medical treatment of patients with uncomplicated AADB demonstrates a lower early mortality risk but with a worse long-term outcome. In complicated patients, surgical treatment bears a higher mortality risk in the acute stage of disease, but the long-term medical prognosis is better. The presence of certain clinical (relapsing pain and uncontrolled hypertension in the first 24 hours) and diagnostic (maximal diameter of the descending aorta over 4.75 cm, the maximum diameter of the abdominal aorta over 3.35 cm) signs at the initial presentation and symptoms (syncope at the initial presentation and the development of intrahospital complications during 24 hours) suggest a higher risk of early mortality in patients with AADB. The most important independent predictors of late mortality are the following clinical signs: relapsing pain and uncontrolled hypertension in the first 24 hours, and pathologic differences in arterial blood pressure over 20 mmHg.

Considering that endovascular therapeutical procedures require financial costs and trained staff, they are not available in all of the cities in the countries that they are being applied to. We think that our data in cases of treatment patients with AADB, medically or surgically, would be significant for doctors that engage in this field of medicine.

Knowing the mortality predictors in AADB patients may help select optimal treatment options. Additional investigations further clarifying the issues assessed here are warranted.

## Figures and Tables

**Table 1:** Baseline characteristics for all patients with type B aortic dissection.

Variable	*N*	%
Age-mean (±SD), year	62.32 (9.62)	—
Gender-male	71	68.3
Any pain reported	103/104	99.0
Abrupt onset of pain	94/103	91.3
Relapsing pain	36/103	34.9
Prior aortic aneurysm	9	8.6
Prior aortic dissection	1	1.4
Prior cardiac Cath/PTCA/CABG	7	6.7
Marfan's syndrome	1	1.4
Hypertension	98	94.3
Treatment of prior hypertension	72	69.7
Uncontrolled hypertension	89/104	85.6
Diabetes	9	8.6
Smoking	61	58.6
Hyperlipidemia	40	38.5
Arterial blood pressure difference over 20 mmHg	31/104	29.8
Syncope	24/104	23.1
Any pulse deficit	34/104	32.7
Monoplegia/monoparesis	11/104	10.5
Cardiac failure	3/104	2.9
Hypotension/shock	3/104	2.9
Auscultated murmur of aortic insufficiency	25/104	24.1
Acute renal failure	15/104	14.4
Neurological symptoms and signs (CVI, spinal ischemia)	10/104	9.6

*N*, number of patients; SD, standard deviation; PTCA, Percutaneous transluminal coronary angioplasty; CABG, Coronary artery bypass graft surgery; CVI, cerebrovascular insult.

**Table 2:** Predictors of in-hospital mortality in patients with acute aortic dissection type B

Variable	HR	95% CI	*p* value
Cox's univariant analysis			
All in-hospital complications	21.20	2.71–26.90	0.003
Relapsing pain	20.43	2.58–26.16	0.004
Uncontrolled hypertension	13.90	2.99–64.59	0.001
Unthrombosed false lumen	8.76	1.12–68.45	0.038
Syncope	4.41	1.34–14.48	0.014
Maximum diameter of dissected descending aorta >4.75 cm	1.71	1.18–2.48	0.004
Maximum diameter of dissected abdominal aorta >3.35 cm	1.76	1.24–2.49	0.001

Cox's multivariant analysis			
Uncontrolled hypertension	20.69	2.60–164.62	0.004
Maximum diameter of dissected aorta >4.75 cm	6.30	1.33–29.93	0.020

CI, confidence interval; HR, hazard ratio.

**Table 3:** Predictors of late (overall) mortality in patients with acute aortic dissection type B.

Variable	HR	95% CI	*p* value
Cox's univariant analysis			
Relapsing pain	49.04	6.50–67.00	<0.001
All in-hospital complications	8.88	2.95–26.65	<0.001
CT-aortic branches being affected	7.11	0.99–50.60	0.050
Unthrombosed false lumen	5.09	1.48–17.43	0.009
Prior aortic aneurism	4.97	1.43–17.23	0.011
Blood pressure difference	3.78	1.54–9.27	0.003
Monoplegia/Monoparesis	3.35	1.09–10.27	0.034
Maximum diameter of dissected descending aorta >4.75 cm	2.90	1.18–7.08	0.020
Aortic regurgitation	2.91	1.00–8.49	0.049
Syncope	2.80	1.15–6.82	0.023
Maximum diameter of dissected abdominal aorta >3.35 cm	1.78	1.32–2.40	<0.001
Gender (male)	0.19	0.04–0.83	0.028
Uncontrolled hypertension	0.10	0.04–0.28	<0.001

Cox's multivariant analysis			
Relapsing pain	7.93	1.00–62.87	0.050
Uncontrolled hypertension	7.25	1.89–27.65	0.003
Blood pressure difference over 20 mmHg	5.33	1.45–19.67	0.012

CI, confidence interval; HR, hazard ratio; CT, computed tomography.

## Data Availability

Data are available upon request to the corresponding author.

## Abbreviations

AAD: Acute aortic dissection
AADA: Acute aortic dissection type A
AADB: Acute aortic dissection type B.

## References

[1] Erbel R., Aboyans V., Boileau C. (2014). ESC Guidelines on the diagnosis and treatment of aortic diseases: document covering acute and chronic aortic diseases of the thoracic and abdominal aorta of the adult. The Task Force for the Diagnosis and Treatment of Aortic Diseases of the European Society of Cardiology (ESC). *European Heart Journal*.

[2] Tran T. P., Khoynezhad A. (2008). Current management of type B aortic dissection. *Vascular Health and Risk Management*.

[3] Howard D. P., Banerjee A., Fairhead J. F., Perkins J., Silver L. E., Rothwell P. M. (2013). Population-based study of incidence and outcome of acute aortic dissection and premorbid risk factor control: 10-year results from the Oxford Vascular Study. *Circulation*.

[4] Meszaros I., Morocz J., Szlavi J. (2000). Epidemiology and clinicopathology of aortic dissection. *Chest*.

[5] Hagan P. G., Nienaber C. A., Isselbacher E. M. (2000). The international registry of acute aortic dissection (IRAD): new insights into an old disease. *JAMA*.

[6] Sampson U. K. A., Norman P. E., Fowkes F. G. R. (2014). Global and regional burden of aortic dissection and aneurysms: mortality trends in 21 world regions, 1990 to 2010. *Global Heart*.

[7] Sampson U. K. A., Norman P. E., Fowkes F. G. R. (2014). Estimation of global and regional incidence and prevalence of abdominal aortic aneurysms 1990 to 2010. *Global Heart*.

[8] Sullivan P. R., Wolfson A. B., Leckey R. D., Burke J. L. (2000). Diagnosis of acute thoracic aortic dissection in the emergency department. *The American Journal of Emergency Medicine*.

[9] Ihara T., Komori K., Yamamoto K., Kobayashi M., Banno H., Kodama A. (2013). Three-dimensional workstation is useful for measuring the correct size of abdominal aortic aneurysm diameters. *Annals of Vascular Surgery*.

[10] Evangelista A., Flachskampf F. A., Erbel R. (2010). Echocardiography in aortic diseases: EAE recommendations for clinical practice. *European Journal of Echocardiography*.

[11] Flachskampf F. A., Badano L., Daniel W. G. (2010). Recommendations for transoesophageal echocardiography: update 2010. *European Journal of Echocardiography*.

[12] Groenink M., den Hartog A. W., Franken R. (2013). Losartan reduces aortic dilatation rate in adults with Marfan syndrome: a randomized controlled trial. *European Heart Journal*.

[13] Chiu H. H., Wu M. H., Wang J. K. (2013). Losartan added to beta-blockade therapy for aortic root dilation in Marfan syndrome: a randomized, open-label pilot study. *Mayo Clinic Proceedings*.

[14] Cambria R. P. (2002). Surgical treatment of complicated distal aortic dissection. *Seminars in Vascular Surgery*.

[15] Tsai T. T., Fattori R., Trimarchi S. (2006). Long-term survival in patients presenting with type B acute aortic dissection: insights from the International Registry of Acute Aortic Dissection (IRAD). *Circulation*.

[16] Lasica R. M., Perunicic J., Mrdovic I. (2006). Temporal variations at the onset of spontaneous acute aortic dissection. *International Heart Journal*.

[17] Kobza R., Ritter M., Seifert B., Jenni R. (2002). Variable seasonal peaks for different types of aortic dissection?. *Heart*.

[18] Mehta R. H., Manfredini R., Hassan F. (2002). Chronobiological patterns of acute aortic dissection. *Circulation*.

[19] Arntz H.-R., Willich S. N., Schreiber C., Brüggemann T., Stern R., Schultheiß H. P. (2000). Diurnal, weekly and seasonal variation of sudden death: population-based analysis of 24, 061 consecutive cases. *European Heart Journal*.

[20] Di Eusanio M., Trimarchi S., Patel H. J. (2013). Clinical presentation, management, and short-term outcome of patients with type A acute dissection complicated by mesenteric malperfusion: observations from the International Registry of Acute Aortic Dissection. *The Journal of Thoracic and Cardiovascular Surgery*.

[21] Genoni M., Paul M., Tavakoli R. (2002). Predictors of complications in acute type B aortic dissection. *European Journal of Cardio-Thoracic Surgery*.

[22] Pape L. A., Awais M., Woznicki E. M. (2015). Presentation, diagnosis, and outcomes of acute aortic dissection: 17-year trends from the international registry of acute aortic dissection. *Journal of the American College of Cardiology*.

[23] Khan I. A. (2001). Clinical manifestations of aortic dissection. *J Clin Basic Cardiol*.

[24] Spittell P. C., Spittell J. A., Joyce J. W. (1993). Clinical features and differential diagnosis of aortic dissection: experience with 236 cases (1980 through 1990). *Mayo Clinic Proceedings*.

[25] Fukunaga N., Koyama T. (2016). Evolution of diagnosis and clinical outcomes in acute aortic dissection: data from the International Registry of Acute Aortic Dissection. *Journal of Thoracic Disease*.

[26] Bockler D., Hyhlik-Durr A., Hakimi M., Weber T. F., Geisbusch P. (2009). Type B aortic dissections: treating the many to benefit the few?. *Journal of Endovascular Therapy*.

[27] Fattori R., Cao P., De Rango P. (2013). Interdisciplinary expert consensus document on management of type B aortic dissection. *Journal of the American College of Cardiology*.

[28] Chan S. H., Liu P. Y., Lin L. J., Chen J. H. (2005). Predictors of in-hospital mortality in patients with acute aortic dissection. *International Journal of Cardiology*.

[29] Tsai T. T., Evangelista A., Nienaber C. A. (2007). Partial thrombosis of the false lumen in patients with acute type B aortic dissection. *New England Journal of Medicine*.

[30] Song J. M., Kim S. D., Kim J. H. (2007). Long-term predictors of descending aorta aneurysmal change in patients with aortic dissection. *Journal of the American College of Cardiology*.

[31] Chan Y. C., Clough R. E., Taylor P. R. (2011). Predicting aneurysmal dilatation after type B aortic dissection. *European Journal of Vascular and Endovascular Surgery*.

[32] Park K. H., Lim C., Choi J. H. (2009). Midterm change of descending aortic false lumen after repair of acute type I dissection. *The Annals of Thoracic Surgery*.

